# Comparing the World Psychiatric Association and European Psychiatric Association Codes of Ethics: Discrepancies and shared grounds

**DOI:** 10.1192/j.eurpsy.2024.1748

**Published:** 2024-05-07

**Authors:** Noemi Sansone, Samuel Tyano, Antonio Melillo, Meryam Schouler-Ocak, Silvana Galderisi

**Affiliations:** 1 University of Campania “Luigi Vanvitelli”, Naples, Italy; 2 Department of Psychiatry, Tel Aviv University Medical School, Tel Aviv, Israel; 3 Psychiatric University Clinic of Charité at St. Hedwig Hospital, Berlin, Germany

**Keywords:** death penalty, distributive justice, domestic violence, education, ethical principles, psychiatry

## Abstract

**Background:**

Codes of ethics provide guidance to address ethical challenges encountered in clinical practice. The harmonization of global, regional, and national codes of ethics is important to avoid gaps and discrepancies.

**Methods:**

We compare the European Psychiatric Association (EPA) and the World Psychiatric Association (WPA) Codes of Ethics, addressing main key points, similarities, and divergences.

**Results:**

The WPA and EPA codes are inspired by similar fundamental values but do show a few differences. The two codes have a different structure. The WPA code includes 4 sections and lists 5 overarching principles as the basis of psychiatrists’ clinical practice; the EPA code is articulated in 8 sections, lists 4 ethical principles, and several fundamental values. The EPA code does not include a section on psychiatrists’ education and does not contain specific references to domestic violence and death penalty. Differences can be found in how the two codes address the principle of equity: the EPA code explicitly refers to the principle of universal health care, while the WPA code mentions the principle of equity as reflected in the promotion of distributive justice.

**Conclusions:**

We recommend that both WPA and EPA periodically update their ethical codes to minimize differences, eliminate gaps, and help member societies to develop or revise national codes in line with the principles of the associations they belong to.

Minimizing differences between national and international codes and fostering a continuous dialogue on ethical issues will provide guidance for psychiatrists and will raise awareness of the importance of ethics in our profession.

## Introduction

Since the early days of medicine, the need to regulate medical practice through ethical frameworks has been acknowledged [1]. The mental health care setting has special ethical dilemmas, and psychiatrists encounter ethical challenges somewhat different from those encountered in other areas of medical practice. The peculiarities of these ethical challenges are rooted in the nature of both psychiatric disorders and the therapeutic relationship between psychiatrists and their patients. Promoting self-determination/autonomy versus envisaging the need to protect a person from self-harm is a good example of an ethical challenge that psychiatrists are more likely to face than other medical doctors.

The development of ethical codes in psychiatry started in the 20th century, mainly due to the deinstitutionalization process and the political abuses and crimes committed during World War II and in the following decades in several countries [2-4]. The need for ethics recommendations for psychiatrists was finally recognized in 1973, with the publication of the APA’s “Principles of Medical Ethics with Annotations Especially Applicable to Psychiatry” and the Declaration of Hawaii, the first international declaration dealing with the ethics of psychiatry, presented during the 1977 World Psychiatric Congress in Honolulu [5].

After several revisions and the integration of new documents, in 1983, the World Psychiatric Association (WPA) adopted the Declaration of Hawaii/II, the first international declaration dealing with ethical issues in psychiatry, and in 1996, the Declaration of Madrid. In 2020, during the Virtual General Assembly, the WPA approved its Code of Ethics. The first draft of this document was presented to the WPA General Assembly in Berlin in 2017, and after several revisions, a final version was approved by the WPA Executive Committee in September 2019. The code is articulated in four sections: 1) ethics in the clinical practice of psychiatry, 2) ethics in psychiatric education, 3) ethics in psychiatric research, and 4) ethics in public mental health [6].

The European Psychiatric Association (EPA) was the first regional psychiatric organization to develop an ethical guidance document with the 2013 “Declaration on Quality of Psychiatry and Mental Health Care in Europe.” This document was later expanded by the EPA Committee on Ethical Issues with the “EPA Code of Ethics,” which was approved by the General Assembly in April 2021 [7]. The code is articulated into eight sections: 1) the fundamental values (as formulated in 1979 by Beauchamp and Childress [8]); 2) psychiatrists’ responsibilities; 3) providing individualized care; 4) psychiatrists as researchers; 5) addressing the media; 6) relationship with industry; 7) relationship with third-party payers, and 8) specific situations (torture, selection of sex, assisted suicide).

Changes in international legislation (e.g., the Convention on the Rights of Persons with Disabilities, United Nations, 2006), cultural and technological developments such as the transition toward digital mental health care [9], and a few differences between the EPA and the WPA Code of Ethics, often reflecting unsolved issues and debates in the psychiatric community, may require revisions in the near future.

In this paper, we highlight the differences between the EPA and WPA Codes of Ethics and discuss them in light of the existing evidence as well as relevant guidance papers and position statements released by the two associations.

## Methods

We conducted a content analysis of the EPA and WPA Codes of Ethics, addressing the main key points, similarities, and divergences. The two documents are publicly available and were retrieved from the official websites of the two associations [4,5]. Initially, three authors (N.S., A.M., S.G.) conducted a thorough reading of the documents separately and identified relevant key points with a text-driven approach. For each document, two authors (N.S. and A.M.) separately extracted phrases, sentences, and paragraphs related to each key point; any disagreement was resolved through the involvement of the corresponding author (S.G.). Each key point and the related content was categorized in main thematic areas by the corresponding author (S.G.) based on their conceptual similarity and, subsequently, a side-by-side comparison of the two Codes of Ethics was conducted for each thematic area both individually and, subsequently, through discussions involving the whole group. Final decisions regarding similarities and differences were determined on a consensus-driven approach, and final results were organized in main thematic areas.

## 1. Results

### Fundamental principles

Regarding the fundamental principles of the profession, both the WPA and EPA indicate beneficence, autonomy, and non-maleficence. The WPA code lists two more overarching principles: improving standards of practice and applying expertise to the service of societies, stating that psychiatrists should help the development of the profession and should use their specialized knowledge to promote mental health (Table 1).

**Table 1.** Main differences between the WPA and the EPA Code of Ethics

Topic	WPA Code of Ethics	EPA Code of Ethics
Structure	The code is articulated into 4 sections: Ethics in clinical practice of psychiatry Ethics in psychiatric education Ethics in psychiatric research and publication Ethics in public mental health	The code is articulated in the following sections: Fundamental values Psychiatrists’ roles Individual treatment Psychiatrists as researchers Engagement with the media Relationship with industry Relations with third–party funders Special situations (torture, gender selection, assisted suicide)
Ethical principles	“Overarching principles”: Beneficence Respect for patients’ autonomy Non–maleficence Improving standards of mental health care and psychiatry practice Applying psychiatric expertise to the service of society Emphasis on promoting well–being and human rights of patients; attention to patient’s families and caregivers; guidance on informed consent.	“Fundamental values”: Respect for autonomy Beneficence Non–maleficence Justice Emphasis on awareness, sensitivity, and empathy; reduction of stigma and prohibition of discrimination; importance of providing diagnoses and treatment information
Access to healthcare	Distributive justice as a fundamental principle. Advocacy for fair and equitable allocation of resources for prevention, treatment, and rehabilitation	Explicit obligation to advocate for universal care and fair prevention, care, treatment, and rehabilitation
Standards of clinical practice	Psychiatrists promote the continuing development of their profession and their personal professional development. Clinical practice should be in accordance with accepted standards. The code emphasizes collaboration with colleagues	Psychiatrists ensure that their knowledge and practices are up to date through continuing education; are aware of the best available treatments for their patients in their respective country and maintain therapeutic boundaries. The code does not mention collegiality as a means to promote standards
Discrimination	Psychiatrists oppose all forms of discrimination against persons with psychiatric disorders and avoid behaviors that might promote discrimination	Psychiatrists shall not discriminate on the basis of age, race, ethnicity, nationality, religion, sex, gender, sexual orientation, social standing, criminal background, disability, disease, or political affiliations
Stigma	Psychiatrists should combat stigma in every possible field and should promote initiatives in public health activities	Psychiatrists should pay attention to reduce stigma and discrimination against mental illness in their clinical practice, in research and in the relationship with the media
Coercion, involuntary treatments, and informed consent	Coercive measures as a last resort. Emphasis on seeking informed consent whenever possible. Guidance on impaired capacity cases	Coercive measures as a last resort and when no alternative can provide safety and adequate care. Consensus for treatment should be sought continuously even in involuntary cases
Death penalty and assisted suicide	Psychiatrists should not participate in the administration of death penalty. Caution on endorsing requests for life–terminating treatments; need to examine whether psychopathological conditions drive such requests	No mention of the death penalty. Psychiatrists should not participate in assisted suicide, respecting their duty to protect life
Political abuse of psychiatry	Psychiatrists should not exploit their profession for political purposes or involve themselves in interrogations of political prisoners	Emphasis on not participating in any form of torture or acts forced by authorities
Public mental health	Psychiatrists should contribute to the improvement of public health, advocating for the interests of individuals with mental disorders, participating in public education. Psychiatrists should also promote distributive justice, including fair and equitable allocation of resources for the prevention, treatment, and rehabilitation of psychiatric disorders. Psychiatrists should work to minimize the occurrence of violence within families, aware of the deleterious consequences of emotional and sexual abuse on mental health and well–being	Psychiatrists have the duty to advocate for universal healthcare for all, to promote mental health and well–being in the population. No specific reference to the role of psychiatrist in domestic violence
Psychiatric research	Detailed guidance on research ethics, emphasis on informed consent, safety, and privacy	Fundamental principles of good research practice are mentioned
Relationship with the media and confidentiality	Emphasis on the need to promote accurate information and address stigma, and advocate for distributive justice	Emphasis on accuracy and on preserving dignity of the subject, of the profession, and of people with mental disorders. Adherence to GDPR for data protection
Education and psychiatry	Emphasis on the teacher–student relationship, ethical considerations in involving students in clinical practice	No specific section on education
Relationship with third–party funders	Recommendation to psychiatrists to avoid relationship with third parties that may compromise their primary interests, and to always disclose financial relationships	Psychiatrists must disclose their affiliations with supporting/ collaborating organizations and financial sponsors avoiding any kind of conflicts

According to the WPA Beneficence principle, psychiatrists have the “duty of promoting the well-being of patients, respecting their human rights, providing competent and compassionate medical care with devotion to the interests of their patients,” and basing their clinical practice on both experiential knowledge and up-to-date scientific information. In this regard, the code emphasizes the importance of attention and sensitivity to the needs not only of patients, but also of their families and caregivers, asserting that “optimal clinical care is achieved through collaboration among patients, caregivers, and clinicians.”

Regarding the autonomy principle, the WPA code states that “psychiatrists are especially mindful of respect for autonomy given their statutory role in treating a proportion of their patients compulsorily” and points out that “compulsory treatment may be justified where a less restrictive intervention cannot achieve safe and adequate care; its purpose is ultimately to promote and re-establish patients’ autonomy and welfare.” The WPA code also addresses matters of confidentiality, therapeutic relationships, and informed consent, offering guidance for cases where patients have impaired capacity to make treatment decisions.

The “Non-maleficence” principle addresses the exploitation and abuse of patients, as well as the discrimination, banning any form of harm through medical and non-medical actions. Special attention is also dedicated to the boundaries of the therapeutic and clinical relationship, the behavior toward vulnerable children and adults, and to the political abuse of psychiatry.

The EPA Code of Ethics states that “Psychiatrists should consider the ethical principles of respect for autonomy, beneficence, non-maleficence, and justice,” and underscores the importance of fostering awareness, sensitivity, and empathy toward the patient as an individual, taking into consideration their cultural values and beliefs.

The WPA code does not include justice as an overarching principle. However, in the section “Ethical principles in public mental health,” it explicitly mentions the need for psychiatrists to promote distributive justice by advocating for a fair and equitable allocation of resources for the prevention, treatment, and rehabilitation of psychiatric disorders.

### Standards of clinical practice

As for the duty to promote the standards of mental health care, the WPA code requires that psychiatrists practice in accordance with accepted standards of care and actively contribute to the development of the profession through ongoing collaboration with their colleagues. The EPA code also requires that psychiatrists keep their knowledge and practice up to date through continuing education and are always informed about the best available treatments in their countries. The code, however, does not mention the issue of collegiality and relationships with colleagues as a means to promote the standards of mental health, as addressed in the WPA code. Both codes dedicate articles to the subject of individualized care and emphasize the importance of providing not only the best available treatment but also the most suitable one based on the patients’ needs and preferences.

### Coercion, involuntary treatments, and informed consent

Both the EPA and WPA acknowledge that coercive measures should be considered only when no alternative action can provide adequate care. However, the EPA code adds that such measures should only be implemented when there is a tangible risk to the patient’s safety or the safety of others. The topic is also addressed in other parts of each code: the WPA code deals with informed consent and involuntary measures in the paragraphs relevant to the autonomy principle, stating that “psychiatrists [should] seek the informed consent of their patients whenever possible. When family members or guardians have authority to make decisions on patients’ behalf, psychiatrists engage them in the process of obtaining informed consent within the local frameworks of confidentiality.” Furthermore, the WPA code recommends that “Psychiatrists will avoid coercing patients regarding their decisions about medical interventions as much as possible.” However, terms and boundaries that psychiatrists might refer to are difficult to define and depend on many variables, including local legislation, training, and resources. Similarly, the EPA code addresses the topic of informed consent as a means to guarantee self-determination and protect patient’s autonomy, stating that “informed consent from patients for care, treatment, rehabilitation, and research is desirable” and when a patient is involuntarily treated, “consensus for treatment should be sought continuously.”

### Death penalty and assisted suicide

Only the WPA code suggests specific conduct regarding death penalty circumstances, stating that psychiatrists must never participate in the administration of such practices. The EPA code does not dedicate a section to this topic, probably because only one of the EPA member associations (Belarusian Psychiatric Association) legally recognizes capital punishment as a penalty.

The two codes also address the topic of assisted suicide in a similar way: the EPA code states that “psychiatrists should treat the illness […] and it is not a psychiatrist’s duty to take part in assisted suicide.” The WPA code states that “psychiatrists avoid endorsing patients’ requests for implementing the termination of life-sustaining treatment or physician-assisted death when they recognize that underlying psychopathology drives those requests.”

### Political abuse of psychiatry

Both codes strongly affirm that psychiatrists should not exploit their profession for political purposes. The EPA code refers to torture specifically, requesting that “Psychiatrists must not take part in any action involving mental or physical torture, even when authorities attempt to force their involvement in such acts.” Similarly, the WPA code states that psychiatrists should not participate or assist in interrogations of political prisoners or collaborate for the detection of anti-government ideas or political or religious prosecutions.

### Psychiatric research

On the topic of ethics in psychiatric research, both the WPA and EPA codes indicate the main criteria that a psychiatrist should respect. The WPA code dedicates an extensive section to the topic and states that when assuming the role of teacher or educator, psychiatrists should recognize their position as role models and that of trainees as vulnerable individuals, and act accordingly. They should promote accurate scientific knowledge and advocate for equity and respect for human rights. The research section discusses extensively the ethical principles that should guide research, stating that “in their roles as researchers and authors, psychiatrists give particular emphasis to the principles of beneficence, non-maleficence, and respect for patients, equity, and for applying psychiatric expertise to the service of society.” Special attention must be paid to research when it involves human volunteers and reaffirms the Nuremberg principle that “research that is unlikely to produce valid results is inherently unethical” [10].

The EPA code simply states that good research practice entails ensuring beneficence, non-maleficence, integrity, informed consent, and respect for people’s rights and dignity.

### Relationship with the media and confidentiality

The WPA code requests psychiatrists to provide accurate information and dispel misconceptions about psychiatric disorders. The WPA code also establishes the duty to actively participate in promoting public mental health by raising awareness, addressing stigma, and, importantly, advocating for distributive justice and ensuring equitable allocation and access to resources for the prevention, treatment, and rehabilitation of psychiatric disorders. The WPA code also refers to psychiatrists’ duty to respect confidentiality in the paragraphs dealing with the autonomy principle and the one relevant to the application of psychiatrists’ expertise to the service of society.

The EPA code also recommends accuracy and stresses that psychiatrists should “conduct themselves and present information in a way that will preserve the dignity of psychiatry as a profession, of mental health care professionals, of patients and of all subjects and topics relevant to psychiatry.” The EPA code also includes a paragraph on confidentiality and the obligation to combat stigma, referring to national laws and the general data processing regulation (GDPR) in the European Union, the main European regulation law on data protection and privacy, which enhances individuals’ control and rights over their personal data.

### Education and psychiatry

The WPA code dedicates a section to ethics in psychiatric education, dealing with the teacher-student relationship and its boundaries, the involvement of students in clinical practice, always keeping in mind the primary goal of caring for the patients. The EPA code does not include a section on education.

### Relationship with industry and third parties

The EPA code recommends that psychiatrists disclose affiliations and financial conflicts of interest, and “ensure that any incentives from sponsors do not influence their professional work and, in turn, the health of their patients.” The WPA code also demands disclosure of financial conflicts of interest, but more explicitly dictates that psychiatrists should avoid relationships with third parties that may influence their primary interests.

## 2. Discussion

In this paper, we highlight and discuss differences and similarities between the Code of Ethics of the World Psychiatric Association and of the European Psychiatric Association. As discussed in the previous paragraphs, these two documents are inspired by similar fundamental values but show a few differences. Some of these differences can be explained through the lens of heterogeneous social, cultural, political, and historical backgrounds. The sections on the political abuse of psychiatry and psychiatrists’ participation in the death penalty, interrogation, detention, and torture are a good example. In fact, the WPA code dedicates more extensive attention to these issues, as compared to the EPA code. This difference might be related to the historical context of abuses of psychiatry that occurred worldwide and still occur, especially outside of Europe [11]; however, an alignment of the two codes on this topic should be considered.

The two codes deal with the principle of equity in access to health care differently. The EPA code refers to the principle of universal health care, currently in effect, although in different forms, in most European countries, while the WPA takes a somewhat broader approach by clearly mentioning the duty to promote “distributive justice,” including (but not limited to) “equitable allocation of resources for the prevention, treatment, and rehabilitation of psychiatric disorders,” thus emphasizing the importance of a wider principle of social and economic justice in the light of its impact on mental health care. This aspect had also been addressed before in the WPA Position Statement on “Social Justice for Persons with Mental Illness” [12], where the WPA highlighted the consequences of economic distress and poverty on mental health. Indeed, there is an overwhelming evidence of the bidirectional relationship between mental health conditions and lower socio-economic conditions as well as homelessness [13-17], and the current literature clearly shows that individuals with mental health conditions, particularly those characterized by an early onset and/or poor premorbid functioning, have an enduring educational gap with respect to the general population [18]. In conclusion, the WPA’s mention of distributive justice and allocation of resources has the advantage of recognizing the deep and complex relationship between socioeconomic factors and mental health, and of clearly acknowledging the beneficial clinical effects of social, economic, and educational interventions [19-23].

There are differences between the two codes of ethics in their relationship with the media. The EPA Code of Ethics regards the preservation of the dignity of psychiatry and people with psychiatric conditions as a duty of psychiatrists. The topic is extremely important, as psychiatrists’ involvement with the media could be against the principles of accuracy, dignity, but also beneficence, non-maleficence, and respect for the person, given the potentially harmful effects on the individual who is the object of the public discussion [24]. The key role of international psychiatric associations’ codes of ethics becomes evident in the light of a recent study that systematically reviewed the topic of psychiatrists’ involvement with the media coverage of mental health issues in different European countries and reported that a sizeable proportion of national psychiatric association did not offer guidance on this specific topic [25]. Therefore, given the importance of communication, especially in the digital era [26], both the EPA and WPA codes might benefit from a revision of the sections relevant to this topic.

A third important difference is the absence of a specific section in the EPA Code of Ethics addressing the topic of ethics in education and the potential conflicts between the interests of psychiatrists as teachers, educators, or mentors, and those of trainees. In relation to the conflicts of interest and the relationships with third parties and pharmaceutical industries, the two codes show a partial discrepancy, as the WPA Code of Ethics more explicitly dictates that psychiatrists should avoid relationships that may influence their primary interests, while the EPA code demands to “ensure that any incentives from sponsors do not influence their professional work” without explicitly indicating the avoidance or the termination of potentially conflicting relationships as the necessary solution. On these topics, both the EPA [27], and more recently, the WPA [28], ratified documents specifically dedicated to this topic.

Last but not least, the WPA code, in the section dealing with ethical principles in public mental health, underscores the importance of minimizing the occurrence of violence within families, aware of its deleterious consequences of emotional and sexual abuse, especially on women and children. The EPA code, on the contrary, does not address the role of psychiatrists in domestic violence.

The WPA and the EPA code of ethics share a common characteristic, that is, a supra-national intended purpose of use, that often leads to the recommendation to act and practice according to the local legislation, and overlooks differences in social and cultural contexts, available resources, and the many factors that may vary drastically from one country to another. Unfortunately, to our knowledge, only 15 of the 145 psychiatric societies members of the WPA have developed national codes of ethics, while the remaining member societies invite their members to rely either on the general medical association’s codes or on the WPA code [3], and only 8 of the 31 EPA member societies participating in a recent survey had their own national code of ethics, while 12 briefly addressed ethical issues in their general mission statement [29].

In conclusion, we recommend that WPA and EPA, in addition to providing periodical revisions of their respective codes of ethics, periodically renew the invitation to their member societies to develop national codes of ethics complying with the principles of the international associations they participate in, while guiding their members through the specificity of each legislation and socio-cultural context. To avoid difficulties for psychiatrists all over the world, and especially for those whose national associations are members of both WPA and EPA, it is advisable that national and international codes of ethics minimize differences and avoid major discrepancies. To this aim, it is important to favor a constant dialog among national and international associations. Medical schools and residency curricula, as well as continuous medical education activities and main national and international conferences, should update their educational content with the goal of promoting awareness of the ethical principles of the medical profession and of the existing ethical codes. Both national and international associations should promote empirical studies identifying ethical conflicts in clinical settings as well as the societal, institutional, organizational, and resource barriers that impede adherence to ethical codes.
